# An Optimization Method for Condition Based Maintenance of Aircraft Fleet Considering Prognostics Uncertainty

**DOI:** 10.1155/2014/430190

**Published:** 2014-04-17

**Authors:** Qiang Feng, Yiran Chen, Bo Sun, Songjie Li

**Affiliations:** ^1^School of Reliability and Systems Engineering, Beihang University, Beijing, China; ^2^Sichuan jiuzhou Aerocont Technologies Co. Ltd., Mianyang, China

## Abstract

An optimization method for condition based maintenance (CBM) of aircraft fleet considering prognostics uncertainty is proposed. The CBM and dispatch process of aircraft fleet is analyzed first, and the alternative strategy sets for single aircraft are given. Then, the optimization problem of fleet CBM with lower maintenance cost and dispatch risk is translated to the combinatorial optimization problem of single aircraft strategy. Remain useful life (RUL) distribution of the key line replaceable Module (LRM) has been transformed into the failure probability of the aircraft and the fleet health status matrix is established. And the calculation method of the costs and risks for mission based on health status matrix and maintenance matrix is given. Further, an optimization method for fleet dispatch and CBM under acceptable risk is proposed based on an improved genetic algorithm. Finally, a fleet of 10 aircrafts is studied to verify the proposed method. The results shows that it could realize optimization and control of the aircraft fleet oriented to mission success.

## 1. Introduction

Prognostic and health management (PHM) technology has a rapid development and been widely used in aeronautical equipment in recent years. The failure position and remain useful life (RUL) of equipment could be predicted by PHM. Further, it can be used in aircraft condition based maintenance (CBM) [1]. However, due to the uncertainty of prognostics, there are certain risks in the maintenance decisions based on the prediction of RUL [2, 3].

The aircraft usually performs mission in fleet manner and shares limited support resource. So, there will be a tradeoff range for fleet CBM. This means each aircraft can choose strategy among dispatching strategy, standby strategy, and maintenance strategy or their combination when the RUL has been obtained, and the synthetic strategy for fleet (combination of each aircraft's strategy) should meet the mission requirement.

There are three forms of RUL in PHM, and each form includes some uncertainty. First is the point value of the time of potential failure. Second is the interval value of the time of potential failure [4–7]. Third is the RUL distribution of the device [8–12]. The third form has the maximum information and the highest availability but is the most difficult in acquisition and application.

Two methods can be used in reducing the impact of prognostics uncertainty on CBM decision. One is to reduce the uncertainty of failure prediction directly so that the decision risk will decrease [13–15]. The other one is to take the prediction uncertainty into account and make the optimum decision under acceptable risk [16–21]. Because the uncertainty of failure prediction could not be completely eliminated, the latter is more useful in engineering applications.

Most research about RUL for CBM is about the life cycle maintenance optimization decisions on single aircraft and researchers would rather consider the maintenance decisions than think about the mission requirements and the dispatched strategy. The research on fleet CBM oriented to mission successes is few. Agent technology and the heuristic algorithm are used to fleet CBM in article [22, 23], but sample point values of RUL were used only.

An optimal aircraft fleet CBM method for aviation unit maintenance is proposed in the paper considering dispatch, mission, and resource constraints. Moreover, the RUL distribution of the key LRM has been transformed into the failure probability of the aircraft, and the calculation method of the costs and risks for mission is given. Then, an optimization decision making method for fleet dispatch and CBM under acceptable risk is proposed based on an improved genetic algorithm.

## 2. Analysis of Aircraft Fleet CBM

### 2.1. Basic Process Analysis

**Figure 1:**
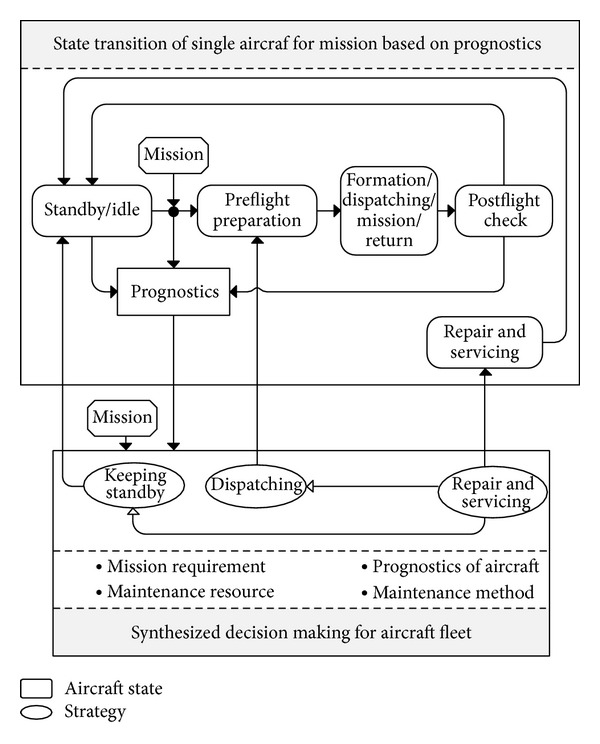
CBM for aircraft fleet.

Consider an aircraft fleet containing *m* aircrafts and *k* integrated support stations (ISS) facing continuous combat missions (*k* < *m*), in which a single mission requires *l* aircrafts (*l* ≤ *m*). Each aircraft contains *p* LRMs of which RUL can be estimated. The mission preparation period starts at time *t*_0_, while the mission period is from time *t*_1_ to *t*_2_. The basic process of the fleet CBM decisions, which is mission success oriented, is given in Figure 1. There are two kinds of single strategies (keeping standby and dispatching) and two kinds of mixed strategies (dispatching after maintenance and standby after maintenance) before making synthesized decision. The fleet CBM decisions consist of these single strategies that should meet the requirements of missions, cost, and risk.

### 2.2. Assumptions

The basic assumptions of the problem are listed below in order to define the problem.

1. The aircraft fails when any key LRM fails.
2. The RUL distribution of LRM which is given at the time *t*_0_ is *F*(*t*) of which probability density function is *f*(*t*).
3. Assume the maintenance method of the LRM is renew, which means the LRM will be as good as new after maintenance, considering the field maintenance of aviation unit maintenance.
4. Only one aircraft can be repaired in each ISS simultaneously. But the total number of the aircraft maintenance may be more than one from the time *t*_0_ to *t*_1_.
5. Different LRM in the same aircraft can be replaced at the same time for renew is served as a maintenance method.
6. The maintenance cost of the different LRM varied while the same LRM cost is the same. The maintenance cost of the LRM on *j* class is *C*_*j*_.
7. Each aircraft malfunction will cause the mission to fail when the fleet is on mission. The consequences of the economic loss will not be taken into consideration.
8. Spare parts are plentiful.

## 3. Modeling Method to Aircraft Fleet CBM Considering Prognostics Uncertainty

### 3.1. Modeling Framework

**Figure 2:**
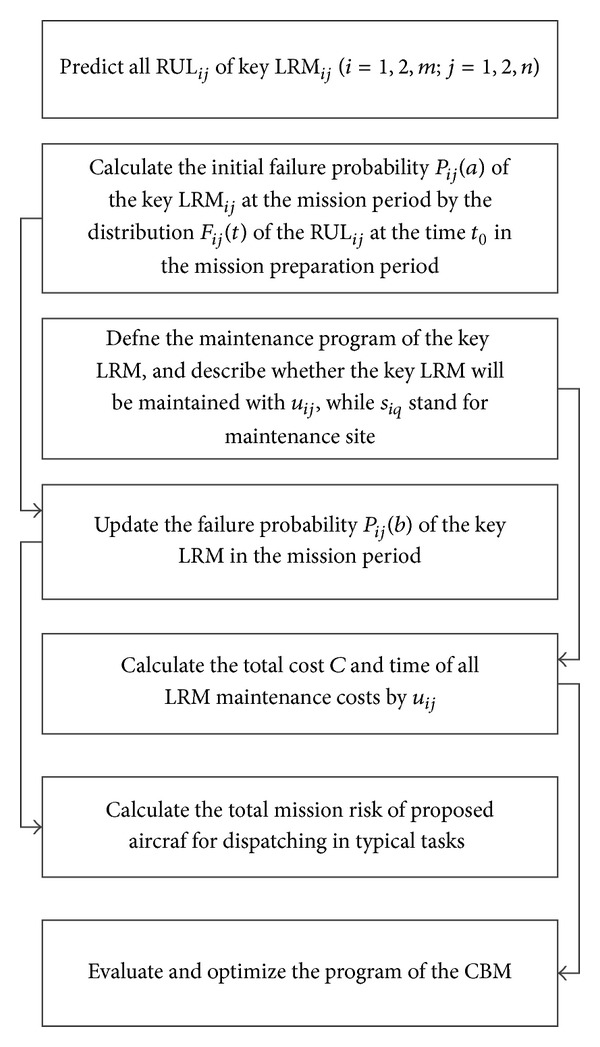
Modeling framework for CBM of aircraft fleet.

The main work of the optimization decision making method for fleet CBM considering prognostics uncertainty includes the following steps: (1) the definition of the fleet initial health status based on the RUL prognostic, (2) maintenance program generating, (3) maintenance time and cost estimation, and (4) mission risk assessment. Based on the objects above, the optimal CBM and maintenance program through the rational optimization algorithm is obtained in the paper. The modeling framework is given in Figure 2.

### 3.2. The Definition of the Fleet Initial Health Status Based on the RUL Prognostic

Assume the distribution of the *j*th key LRU_*ij*_ on the *i*th aircraft is *F*_*ij*_(*t*). In the mission period (*t*_1_-*t*_2_), the probability of failure can be got by

(1) $$\begin{array}{c} p_{ij}\left(a\right)=\overset{t_{2}}{\underset{t_{1}}{\int }}f_{ij}\left(t\right)dt\;i=1,2,\ldots ,m\;\;j=1,2,\ldots ,n, \end{array}$$

where *f*_*ij*_(*t*) is the probability density function of the *F*_*ij*_(*t*).

Considering an aircraft fleet containing *m* aircrafts and each include *n* LRMs, the initial health status matrix of all LRM is *P*(*a*) that is given by

(2) $$\begin{array}{c} P\left(a\right)={\left[\begin{array}{ccc} p_{11}\left(a\right) & \cdots & p_{1n}\left(a\right)\\ \cdots & \cdots & \cdots \\ p_{m1}\left(a\right) & \cdots & p_{mn}\left(a\right) \end{array}\right]}_{m\times n}. \end{array}$$

### 3.3. Maintenance Program Generating

Maintenance program considers whether a certain LRM should be maintained and the selection of the ISSs.

The maintenance matrix *U* of fleet can be described as

(3) $$\begin{array}{c} U={\left[\begin{array}{ccc} u_{11} & \cdots & u_{1n}\\ \cdots & \cdots & \cdots \\ u_{m1} & \cdots & u_{mn} \end{array}\right]}_{m\times n}, \end{array}$$

where *u*_*ij*_ = 1 means that the *j*th LRM of the *i*th aircraft needs to be repaired; otherwise, *u*_*ij*_ = 0.

According to the assumption (4), the LRM can be maintained at the same place; however, many the LRMs fails. Therefore, the ISS matrix *S* of the fleet is shown as

(4) $$\begin{array}{c} S={\left[\begin{array}{ccc} s_{11} & \cdots & s_{1k}\\ \cdots & \cdots & \cdots \\ s_{m1} & \cdots & s_{mk} \end{array}\right]}_{m\times k}, \end{array}$$

where *s*_*iq*_ = 1 means that the *i*th aircraft should be maintained at the *q*th ISS; otherwise, *u*_*ij*_ = 0.

### 3.4. Maintenance Time and Cost Estimation

Assume repairing the *j*th LRM spends time *T*_*j*_ and needs cost *C*_*j*_. According to the assumption (5), the total maintenance time *Tm*_*i*_ of the *i*th aircraft is given as

(5) $$\begin{array}{c} Tm_{i}=\max\left(u_{ij}\times T_{j}\right),\;j=1,2,\ldots ,n. \end{array}$$

There may be more than one aircraft that should be repaired at ISS *q*, so the total maintenance time of all aircrafts can be calcluated as

(6) $$\begin{array}{c} \overset{m}{\underset{i=1}{\sum }}s_{iq}\times Tm_{i}\;i=1,2,\ldots ,m. \end{array}$$

The total maintenance cost of all aircrafts can be got as

(7) $$\begin{array}{c} C=\overset{m}{\underset{i=1}{\sum }}\overset{n}{\underset{j=1}{\sum }}\left[u_{ij}\times C_{j}\right]\;i=1,2,\ldots ,m\;\;j=1,2,\ldots ,n. \end{array}$$

### 3.5. Mission Risk Assessment

**Step 1 (modify the health matrix of the fleet)**

Whether the aircraft is “dispatching” or “dispatching after maintenance” should be taken into consideration when calculating the mission risk of the fleet. The status of the aircraft should be updated if the single strategy of the fleet is “dispatching after maintenance.” Then, the modified health status matrix *P*(*b*) of the fleet can be built according to the Assumption (3). Consider *p*_*ij*_(*b*) = 0 after the LRM_*ij*_ on the *i*th aircraft was renewed; otherwise, *p*_*ij*_(*b*) = *p*_*ij*_(*a*) without renew. The elements in the matrix can be obtained by

(8) $$\begin{array}{c} p_{ij}\left(b\right)=p_{ij}\left(a\right)\times \left(1-u_{ij}\right). \end{array}$$

**Step 2 (estimate the failure probability of the single aircraft and rank)**

The failure probability of the single aircraft could be estimated after modifying the health matrix of the fleet. According to the first assumption, “the aircraft fails when any key LRM fails”; the failure probability *P*_*i*_(*a*) of the *i*th aircraft can be given as

(9) $$\begin{array}{c} P_{i}\left(a\right)=1-\overset{n}{\underset{j=1}{\prod }}\left[1-p_{ij}\left(b\right)\right]. \end{array}$$

Formula (10) can be obtained according to (1), (8), and (9):

(10) $$\begin{array}{c} P_{i}\left(a\right)=1-\overset{n}{\underset{j=1}{\prod }}\left[1-\overset{t_{2}}{\underset{t_{1}}{\int }}f_{ij}\left(t\right)\left(1-{u_{i}}_{j}\right)\right]\;i=1,2,\ldots ,m. \end{array}$$

Then, Pro(*i*) = 1,2,…, *m* can be obtained by sorting the failure probability of single aircraft in ascending order. The ordered failure probability of the aircraft is given as

(11) $$\begin{array}{c} P_{\text{pro}(i)}\left(b\right)=P_{i}\left(a\right). \end{array}$$

Suppose *P*_2_(*a*) is the smallest Pro(*i*). Then, set Pro(2) = 1, and let *P*_1_(*b*) = *P*_2_(*a*) after reordering. Pick up aircrafts of which Pro(*i*) = 1,2,…, *l* when the mission needs dispatch *l* aircrafts.

**Step 3 (calculate the mission risk of the fleet)**

Assume the serious consequences of the mission that failed are similar without taking the economic losses into account. The failure probability of the fleet mission can be calculated by the following according to (7):

(12) $$\begin{array}{c} P_{F}=1-\overset{l}{\underset{i=1}{\prod }}\left[1-P_{i}\left(b\right)\right], \end{array}$$

where *P*_*F*_ is the mission risk.

## 4. Optimization Problem and Algorithms Design

### 4.1. Problem Description

The optimization problem in the paper is to find a fleet CBM strategy with acceptable risk and lowest cost considering prognostics uncertainty.

Thus, describe the objective of the optimization as *Min*⁡*C* = ∑_*i*=1_^*m*^∑_*j*=1_^*n*^[*u*_*ij*_ × *C*_*j*_].

The constraints that should be considered about involve the maintenance ability constraint *R*_*A*_ of the site, the time constraint *R*_*B*_, the security risk constraint *R*_*C*_, the mission risk constraint *R*_*D*_, and the variable constraint *R*_*E*_.

For first constraint *R*_*A*_, set *s*_*iq*_ = 0  (*q* = 1,2 …, *k*) and *u*_*ij*_ = 0  (*j* = 1,2 …, *n*) if none of LRMs need maintenance. Else if any LRM_*ij*_ requires maintenance, then *u*_*ij*_ = 1 and the corresponding *s*_*iq*_ = 1 while the other *s*_*iq*_ = 0. Thus, the *R*_*A*_ can be described as

(13) $$\begin{array}{c} R_{A}:\overset{k}{\underset{q=1}{\sum }}s_{iq}+\overset{n}{\underset{j=1}{\prod }}\left(1-u_{ij}\right)=1. \end{array}$$

All maintenance of site *q* should be finished before the mission starts. Thus, the *R*_*B*_ can be given as

(14) $$\begin{array}{c} R_{B}:Tm_{i}\leq t_{1}-t_{0}\;|\;s_{iq}=1\;i=1,2,\ldots ,m\;\;q=1,2\ldots ,k. \end{array}$$

Assume that the total number of the aircraft which maintained at site *q* is ∑_*i*=1_^*m*^ *s*_*iq*_ = *x* > 1 (where the number of aircraft is 1,2,…, *x*). If ∑_*i*=1_^*x*−1^ *s*_*iq*_ × *Tm*_*i*_ ≤ *t*_1_ − *t*_0_ < ∑_*i*=1_^*x*^ *s*_*iq*_ × *Tm*_*i*_, which means the maintenance for the *x*th aircraft could not be finished before the mission start time, this aircraft will not be taken into account when the maintenance decisions is “dispatching after maintenance.”

It will not be allowed to dispatch if the failure probability is too high for security risk existing in single aircraft. Consider a mission need *l* aircrafts, and the *R*_*C*_ can be described as (15). Moreover, the mission will fail if (15) could not be met. We have

(15) $$\begin{array}{c} R_{C}:P_{l}\left(b\right)<P_{sl}. \end{array}$$

According to (12), *R*_*D*_ can be written as (16) considering the mission risk for fleet. We have

(16) $$\begin{array}{c} R_{D}:P_{F}=1-\overset{l}{\underset{i=1}{\prod }}\left[1-P_{i}\left(b\right)\right]<P_{m}, \end{array}$$

where *P*_*m*_ is the objective of the mission risk.

The variable constraint, which means that the variables should be in a certain range, is described as

(17) $$\begin{array}{c} R_{E}:C_{j}>0,\;\;u_{ij}\in \left\{0,1\right\},\;\;s_{iq}\in \left\{0,1\right\}\\ i=1,2,\ldots ,m\;j=1,2,\ldots ,n\;q=1,2\ldots ,k. \end{array}$$

The conceptual model for aircraft fleet condition based maintenance and dispatch is given as follows:

(18) Min⁡ C=∑i=1m∑j=1n[uij×Cj]  s.t.  RA~E  is  satisfied.

### 4.2. Optimization Algorithms Design

The optimization problem cannot meet the KKT (*Karush-Kuhn-Tucker*) conditions, and the dimension of decision making variables which can be written as *m* × *n* + *m* × *k* is relatively large. So an improved genetic algorithm was proposed in this paper for the problem instead of traditional mathematical methods.

The optimization model can be simplified as

(19) min⁡ C(U,S)  s.t. {g(U,S)≤0h(U,S)=0uij∈{0,1}sij∈{0,1}},

where *U* is the maintenance matrix while the *S* is the ISS matrix.

**Figure 3:**
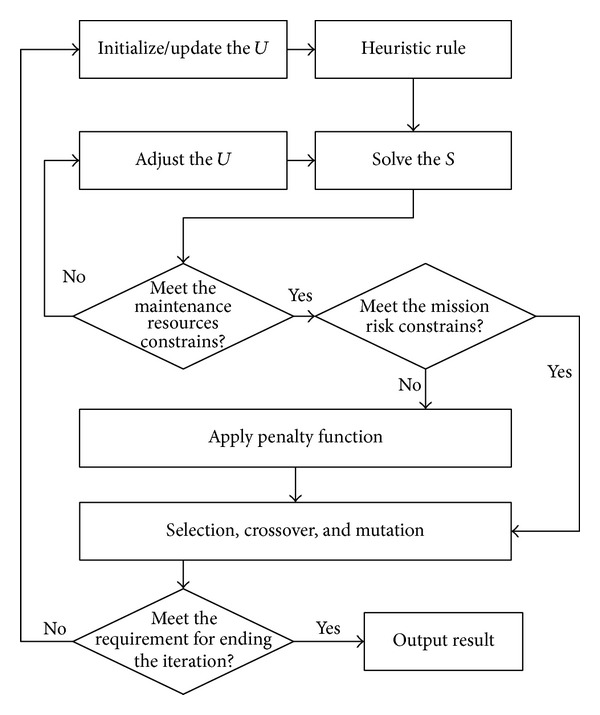
Improved strategies for genetic algorithm.

The problem has more variables and constraints, so the solution quality of problem and the convergence rate could not be satisfied. Therefore, the improvement strategy of the genetic algorithm is given in Figure 3.

#### 4.2.1. Define the Initial Population of the Maintenance Matrix *U*

According to the multifactor and 2-level orthogonal experimental design in order to cover widely, define the initial population of the maintenance matrix *U*. The initial population should be filtered so as to make the convergence faster. Moreover, the number of the aircraft needs to be repaired in the population which should be less than *l* considering the dispatched requirements and the cost of the maintenance. The relationship among those factors is shown as

(20) $$\begin{array}{c} \overset{m}{\underset{i=1}{\sum }}\left[\overset{n}{\underset{j=1}{\prod }}\left(1-u_{ij}\right)\right]\geq m-l. \end{array}$$

#### 4.2.2. Solve the ISS Matrix *S*

It is necessary to find a set of feasible solutions which meet the constraint of the ability of ISS *R*_*A*_ and the maintenance time *R*_*B*_ on the basis of a certain *U*. The following heuristic rules can be used in order to reduce the amount of computation, increasing the efficiency of solving.

**Step 1**

According to formula (13), an initial value of the *S* can be given with the certain *U*. If ∏_*j*=1_^*n*^(1 − *u*_*ij*_) = 1, the aircraft *i* need not be repaired and all *s*_*iq*_ (*q* = 1,2,…, *k*) = 0; otherwise, the aircraft *i* needs to be repaired. Then, the determining condition is described as ∑_*q*=1_^*k*^ *s*_*iq*_ = 1 and *s*_*iq*_ ∈ {0,1}.

**Step 2**

Consider that there are*y* aircrafts need not be repaired. Remove *y* rows which stand for these aircrafts. Then, a new (*m* − *y*) × *k* matrix *S*′ which represents the new relationship between the ISS and the aircraft that needs to be repaired can be built as the reduced cycle matrix of *S*.

**Step 3**

The maintenance time *Tm*_*i*_ of the aircraft needs to be repaired in matrix *S*′ which can be obtained by formula (5). Then, the average maintenance time AMT of the ISSs is given by AMT = ∑_*i*=1_^*m*−*y*^ *Tm*_*i*_/*k*. It can be determined not meet the time constrain if max⁡(*Tm*_*i*_) > *t*_1_ − *t*_0_ or AMT > *t*_1_ − *t*_0_, then turn to Step 6. Otherwise, turn to Step 4.

**Step 4**

Initialize the matrix *S*′, and set *s*_*iq*_ = 0 (*i* = 1,2,…, *m* − *y*, *q* = 1,2,…, *k*).

**Step 5**

Set the value of the matrix *S*′ from the first row to the *k*th row. The method of the *q*th is described as follows.

- Calculate the value of |*Tm*_*i*_ − AMT|  (*i* = 1,2,…, *m*-*y*). If the aircraft *z* makes the |*Tm*_*z*_ − AMT | = min⁡|*Tm*_*i*_ − AMT|, then *s*_*zq*_ = 1. Furthermore, the aircraft can be selected in random if there is more than one aircraft that meets this formula.
- Remove the line in which the aircraft *z* is in to build a new reduced cycle matrix *S*′. Update the remaining maintenance time *TG*_*q*_ = *t*_1_ − *t*_0_ − *Tm*_*z*_ of the site *q*.
- Compare the *TG*_*q*_ and the *Tm*_*i*_ for the *S*′. If the formula “min⁡(*Tm*_*i*_) | *i* = *o* ≤ *TG*_*q*_” can be met by a parameter *o*, form a new reduced cycle matrix and set *s*_*oq*_ = 1. Moreover, the remaining available reference time should be updated as *TG*_*q*_ = *TG*_*q*_(*b*) − *Tm*_*o*_. This work should be repeated until the min (*Tm*_*i*_) > *T*_*G*_ *q*; then, turn to the (*q* + 1)th row.

**Step 6**

The matrix *U* should be adjusted if the constraints of resource maintenance cannot be met. Consider that the maintenance cost should be as low as possible and the requirement of the mission risk should be satisfied; the elements which *u*_*ij*_ = 1 should find *p*_*ij*_(*a*) corresponded and the min⁡{*p*_*ij*_(*a*)}; then, set *u*_*ij*_ = 0. Return to Step 1 and repeat after finishing the update for the *U* until meeting constrains *R*_*A*_ and *R*_*B*_.

#### 4.2.3. Deal with the Constrain of the Mission Risk

Some matrix *U* which is initial or got by adjusting, crossover, and mutation may not meet the requirement of the mission risk constrain. The penalty method can be used in the method followed to solve this problem.

The energy function for every *U* can be written as

(21) $$\begin{array}{c} E\left(U,S\right)=C\left(U,S\right)+F\left(U,S\right)\cdot M^{T}, \end{array}$$

where *F*(*U*, *S*) is the vector of the penalty function and the *F*_*i*_(*U*, *S*) = max⁡{0, *g*_*i*_(*U*, *S*)}, while *M*, which is the penalty factor vector, is a large positive number.

**Step 1 (fitness function design)**

The fitness function is given as follows in order to minimize the objective function:

(22) $$\begin{array}{c} f\left(U,S\right)=1-\frac{E\left(U,S\right)-E_{\min}}{E_{\max}-E_{\min}}, \end{array}$$

where *E*_max⁡_ and *E*_min⁡_ are the maximum and the minimum values of the energy function in the population.

**Step 2 (selection, crossover, and mutation)**

Proportional selection, single-point crossover, and the basic alleles can be used in solving this problem.

This problem can be dealt with by some method written in the article [24–26] in order to avoid the premature and the stalling that appear in the genetic algorithms.

Simulate the annealing stretching for fitness before selecting the operator as follows:

(23) $$\begin{array}{c} f_{i}=\frac{e^{f_{i}/T}}{\sum_{j=1}^{N}e^{f_{j}/T}}\;T=T_{0}\times c^{g-1}\;\;0<c<1, \end{array}$$

where *N* is the size of the population and *g* is the genetic algebra, while *T*_0_ is the initial temperature and *f*_*i*_ is the fitness of the* i*th individual.

*P*_*e*_ and *P*_*f*_ can be defined as (24) in order to make the crossover and mutation probability changing dynamic with the fitness, which means that if the fitness of each individual is consistent, *P*_*e*_ and *P*_*f*_ will increase; otherwise, they will decrease:

(24) $$\begin{array}{c} P_{e}=\{\begin{array}{cc} \frac{k_{1}\left(f_{\max}-f^{\prime }\right)}{\left(f_{\max}-f_{\text{avg}}\right)} & f^{\prime }\geq f_{\text{avg}}\\ k_{2} & f^{\prime }<f_{\text{avg}}, \end{array}\\ P_{f}=\{\begin{array}{cc} \frac{k_{3}\left(f_{\max}-f^{\prime }\right)}{\left(f_{\max}-f_{\text{avg}}\right)} & f^{\prime }\geq f_{\text{avg}}\\ k_{4} & f^{\prime }<f_{\text{avg}}, \end{array} \end{array}$$

where *f*_max⁡_ and *f*_avg_ are the maximum fitness and the average fitness in the populations and the *f*′ is the maximum fitness of the parent. The *k*_1_, *k*_2_, *k*_3_, *k*_4_ are all constant.

## 5. Case Study

Consider a fleet containing 10 aircrafts and each aircraft includes 4 LRM (A, B, C, D) of which life can be predicted. Assume the RUL following Gaussian distributions *N*(*μ*, *σ* ^2^), and the mean *μ* and the variance *σ* ^2^ are given in Table 1.

The mission requires dispatch 8 aircrafts one hour later and lasting two hours. *P*_*sl*_ should be below the 10^−8^ while *P*_*sl*_ should be below 10^−6^.

Assume there are 3 ISSs, of which ability of the maintenance are the same, being in charge of all aircrafts' maintenance. The maintenance time and cost of each LRM is given in Table 2.

Consider that there are 100 individuals in populations, and one of these individuals is described as follows:

(25) $$\begin{array}{c} U={\left[\begin{array}{cccccccccc} 1 & 1 & 0 & 1 & 0 & 0 & 0 & 1 & 1 & 1\\ 0 & 1 & 0 & 0 & 0 & 1 & 1 & 0 & 0 & 0\\ 1 & 0 & 1 & 0 & 1 & 1 & 0 & 0 & 1 & 0\\ 0 & 0 & 0 & 1 & 0 & 0 & 1 & 1 & 0 & 1 \end{array}\right]}^{T}. \end{array}$$

**Figure 4:**
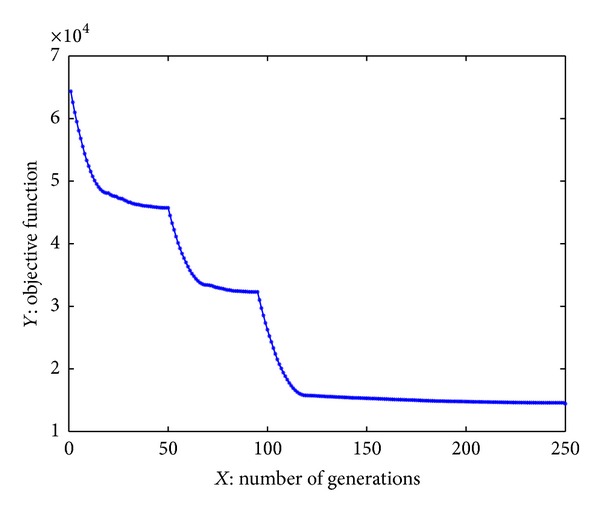
Result of calculation.

Set up the *k*_1_ = *k*_2_ = 0.97, *k*_3_ = *k*_4_ = 0.02. The result is described in Figure 4 after 250 iterations.

The total cost of the maintenance is 14439.3 and the mission risk is 8.95 ∗ 10^−07^ which meet the requirement.

Then, optimal maintenance program can be written as Table 3:

(26) $$\begin{array}{c} U={\left[\begin{array}{cccccccccc} 0 & 0 & 0 & 0 & 0 & 0 & 0 & 0 & 0 & 0\\ 0 & 0 & 0 & 0 & 0 & 1 & 0 & 1 & 0 & 1\\ 1 & 0 & 1 & 0 & 0 & 0 & 0 & 0 & 0 & 0\\ 0 & 0 & 0 & 1 & 0 & 0 & 0 & 1 & 0 & 1 \end{array}\right]}^{T},\\ S={\left[\begin{array}{cccccccccc} 1 & 0 & 0 & 0 & 0 & 1 & 0 & 0 & 0 & 0\\ 0 & 0 & 1 & 0 & 0 & 0 & 0 & 1 & 0 & 0\\ 0 & 0 & 0 & 1 & 0 & 0 & 0 & 0 & 0 & 1 \end{array}\right]}^{T}. \end{array}$$

Then, the optimal scheme of aircraft CBM and dispatching are described completely in Table 3, where the elements in the table such as LRMc, LRM_B/D_ are the LRMs that need to be repaired. There are six aircrafts and eight LRMs that need to be repaired, and the numbers of the aircrafts that need dispatch are 1, 3, 4, 5, 6, 7, 8, and 10.

## 6. Conclusion

This paper researches optimization decision method for aircraft fleet CBM oriented to mission success considering prognostics uncertainty and the resource constrain. The CBM and dispatch process of fleet is analyzed; the modeling method and an improved genetic algorithm for the problem are given, and the method is verified by case about fleet with 10 aircrafts.

The main advantages of this method are shown as follows.

1. The alternative strategy sets for single aircraft are defined; then, the optimization problem of fleet CBM is translated to the combinatorial optimization problem of single aircraft strategy. The relationship between maintenance strategy and mission risk is established, and the problem becomes easier to solve.
2. This paper used the RUL distribution, which has the maximum information and the highest in prognostics. It has more accurate description of the uncertainty compared with others.
3. The optimization decision with risk for fleet CBM is realized. The fleet mission risk is quantitatively assessed, and the optimal CBM strategy for fleet could satisfy the requirement of lowest maintenance cost and acceptable risk.

This paper presents a theoretical approach for fleet CBM considering prognostics uncertainty. Some factors have been simplified, such as the cost of risk, the consequences of risk mission, the effect of the CBM process form ability of maintenance personnel, and the effect of random failures. The focus of further work is a more detailed and comprehensive model considering all above factors.

## Figures and Tables

**Table 1:** The RULs of the LRM.

Number	1	2	3	4	5	6	7	8	9	10
LRM_A_	(25, 7.7)	(19, 4.4)	(29, 8.3)	(29, 9.1)	(13, 3.8)	(20, 5.7)	(21, 6.3)	(20, 6.2)	(9, 2.5)	(21, 5.9)
LRM_B_	(28, 7.4)	(3, 0.7)	(29, 6.6)	(15, 3.3)	(28, 8.5)	(2, 0.5)	(23, 5.6)	(6, 1.3)	(2, 0.5)	(10, 2.8)
LRM_C_	(4, 1.0)	(9, 2.9)	(5, 1.2)	(25, 5.7)	(24, 7.1)	(26, 7.9)	(23, 6.1)	(22, 7.2)	(3, 0.8)	(29, 9.1)
LRM_D_	(28, 7.5)	(17, 5.6)	(30, 7.9)	(5, 1.2)	(29, 7.9)	(29, 7.1)	(12, 3.5)	(1, 0.3)	(25, 6.4)	(2, 0.6)

**Table 2:** The maintenance time of the LRM.

LRM	A	B	C	D
Maintenance time	20 min	25 min	11.6 min	16.6 min
Maintenance cost	2348.2	2843	1297.3	1009.2

**Table 3:** The optimal scheme for aircraft fleet CBM and dispatching.

Aircraft number	1	2	3	4	5	6	7	8	9	10
ISS 1	LRM_C_	/	/	/	/	LRM_B_	/	/	/	/
ISS 2	/	/	LRM_C_	/	/	/	/	LRM_B/D_	/	/
ISS 3	/	/	/	LRM_D_	/	/	/	/	/	LRM_B/D_

Dispatch?	Yes	No	Yes	Yes	Yes	Yes	Yes	Yes	No	Yes
